# Protective Effects of *Peucedanum japonicum* Extract against Osteoarthritis in an Animal Model Using a Combined Systems Approach for Compound-Target Prediction

**DOI:** 10.3390/nu10060754

**Published:** 2018-06-11

**Authors:** Jin Mi Chun, A Yeong Lee, Joong Sun Kim, Goya Choi, Seung-Hyung Kim

**Affiliations:** 1 Herbal Medicine Research Division, Korea Institute of Oriental Medicine, 1672 Yuseong-daero, Yuseong-gu, Daejeon 34054, Korea; lay7709@kiom.re.kr (A.Y.L.); centraline@kiom.re.kr (J.S.K.); serparas@kiom.re.kr (G.C.); 2 Department of Life Systems, Sookmyung Women’s University, Cheongpa-ro 47-gil 100, Yongsan-gu, Seoul 04310, Korea; 3 Institute of Traditional Medicine and Bioscience, Daejeon University, Daejeon 34520, Korea

**Keywords:** *Peucedanum japonicum*, osteoarthritis, monosodium iodoacetate, inflammatory mediator, network pharmacology, compound-target gene network

## Abstract

*Peucedanum japonicum* Thunberg is an herbal medicine used to treat neuralgia, rheumatoid arthritis, and inflammatory-related diseases. However, its effects on osteoarthritis (OA) and its regulatory mechanisms have not been investigated by network analysis. Here, we investigated the pharmacological effects of *Peucedanum japonicum* extract (PJE) on OA, by combining in vivo effective verification and network pharmacology prediction. Rats in which OA was induced by monosodium iodoacetate (MIA) were treated with PJE (200 mg/kg), and histopathological parameters, weight bearing distribution and inflammatory factors in serum and joint tissue were measured after 28 days of treatment. Additionally, in silico network analysis was used to predict holistic OA regulatory mechanisms of PJE. The results showed that PJE exerted potential protective effects by recovering hind paw weight bearing distribution, alleviating histopathological features of cartilage and inhibiting inflammatory mediator levels in the OA rat model. Furthermore, network analysis identified caspase-3 (CASP3), caspase-7 (CASP7), and cytochrome P450 2D6 (CYP2D6) as potential target genes; in addition, the TNF (Tumor necrosis factor) signaling pathway was linked to OA therapeutic action. Our combined animal OA model and network analysis confirmed the therapeutic effects of PJE against OA and identified intracellular signaling pathways, active compounds and target genes linked to its therapeutic action.

## 1. Introduction

Osteoarthritis (OA) is a common degenerative inflammatory joint disease characterized by joint cartilage degeneration, subchondral bone sclerosis, osteophyte formation, and joint tissue inflammation [1]. Although the pathogenesis of OA has not been fully elucidated, OA treatments have been developed to relieve symptoms such as joint pain and stiffness, reduce inflammation, and prevent joint damage [2]. There is currently no cure for OA, and the molecular mechanisms underlying the disease are not yet fully understood. However, there is growing interest in delaying or treating OA using new therapeutic approaches, and many studies highlight the potential of natural products. The causes of OA are multifactorial, and disease progression is highly complex and involves multiple tissues. Thus, herbal medicines and natural products that target multiple mechanistic pathways have great potential as OA treatments.

One herbal medicine attracting attention is *Peucedanum japonicum* Thunberg (PJ), which is used to treat colds, headaches, neuralgia, rheumatoid arthritis and other inflammatory diseases in some Asian countries [3,4]; it also has anti-obesity [5,6], anti-nociceptive [4], anti-osteoporotic [7], and anti-allergic lung inflammatory properties [8]. However, it remains unclear whether PJ is pharmacologically active against OA. The pharmacological effects of PJ have been linked to various components, and phytochemical studies have indicated that the roots contain coumarins, chromones, polyacetylenes, inositols, steroid glycosides, and dihydropyranocoumarins [9]. Several studies report the identities and pharmacological activities of compounds isolated from PJ [10,11,12,13,14].

The network pharmacology approach can be used to investigate drug-target interactions of herbal medicines, and to uncover the underlying molecular mechanisms [15]. This method is attracting attention as a tool for clarifying the molecular mechanisms underlying the effects of herbal medicines on complex diseases such as OA. This approach can consider multiple targets and multiple effects simultaneously, and applying it to complex diseases such as OA could provide insight into the complex mechanisms of herbal medicines [16,17,18]. Thus, the therapeutic effects of PJ on OA and the underlying mechanisms could benefit from network pharmacologybased analysis.

Although various isolated components of PJ and their effects have been investigated, network pharmacology analysis of the effects on OA has not been performed. Therefore, in the present study we investigated the pharmacological effects of PJ extract (PJE) by examining its inhibitory effects in monosodium iodoacetate (MIA)-induced model rats. We then holistically evaluated the regulatory mechanisms of PJE using pharmacological network analysis to identify potential active compounds and OA related target genes.

## 2. Materials and Methods

### 2.1. Preparation of PJE

Roots of PJ were purchased from Kwangmyongdang Co. (Ulsan, Korea) and authenticated based on macroscopic characteristics as described by Dr. Goya Choi of the Korea Institute of Oriental Medicine. A voucher specimen was deposited in the Korean Herbarium of Standard Herbal Resources (Accession no. 2014 PJE-1). The dried herb (289.8 g) was extracted twice with 70% ethanol (by 2 h reflux), and the extract was concentrated as described previously [8]. The decoction was filtered, lyophilized and stored at 4 °C. The yield of the dried extract from the crude material was 34.0% (*w*/*w*). Before use in animal experiments, the lyophilized powder was dissolved in vehicle (0.25% carboxymethylcellulose).

### 2.2. The MIA-Induced OA Rat Model

#### 2.2.1. Animals

Male Sprague-Dawley rats (7 weeks old) were purchased from Samtako Inc. (Osan, Korea) and housed under controlled conditions with a 12-h light/dark cycle at 22 °C ± 2 °C and 55% ± 15% humidity. All animal experiments were performed with the approval of the Institutional Animal Care and Use Committee of Daejeon University (DJUARB-2016-019, 7 June 2016).

#### 2.2.2. Induction of OA and Drug Treatment

**Figure 1:**
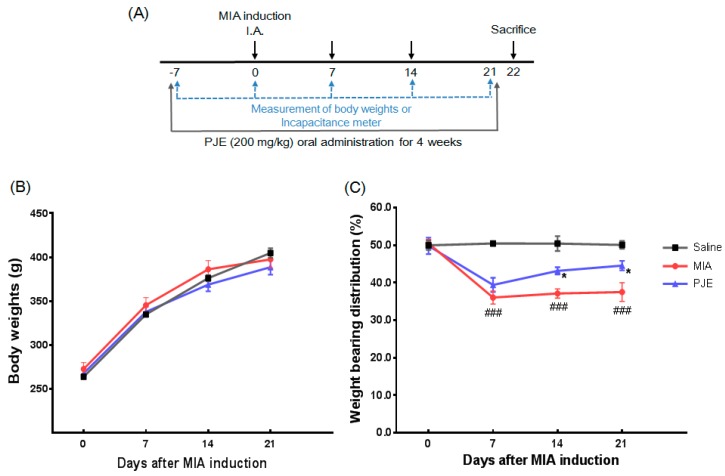
The experimental scheme and the effects of *Peucedanum japonicum* extract (PJE) on the hind paw weight bearing distribution in monosodium iodoacetate (MIA)-induced osteoarthritis (OA) model rats. (**A**) Experimental protocol used to induce osteoarthritis, followed by administration of PJE. I.A., intra-articular injection. (**B**) Body weights and (**C**) weight bearing distribution were measured (once per week for 21 days after injection of MIA) using an incapacitance tester. ### *p* < 0.001 vs. saline controls; * *p* < 0.05 vs. the MIA group.

MIA (Sigma-Aldrich, Germany) was used to induce OA as described previously [19]. Briefly, all groups of rats except the saline (control) group were directly injected with MIA (3 mg in 50 μL of 0.9% saline) into the intra-articular space of the right knee. Rats were orally administered daily with PJE (200 mg/kg) and 1 week before MIA injection for 4 weeks. After 1 week of acclimatization, rats were divided randomly into three groups (*n* = 7 per group) as follows: (1) Saline group (Control): saline alone (no MIA injection); (2) MIA group (MIA + Saline): saline plus MIA injection; and (3) PJE group (MIA + PJE): PJE (200 mg/kg) plus MIA injection. The experimental scheme is shown in Figure 1A.

#### 2.2.3. Measurement of Hind Paw Weight-Bearing Distribution

After OA induction, the weight of rats was measured along with the weight-bearing capability on days 0, 7, 14, and 21. The original balance in weight-bearing capability of the hind paws was disrupted, and an incapacitance tester (Linton Instrumentation, Norfolk, UK) was used to evaluate changes in weight-bearing tolerance. Rats were carefully placed into the measuring chamber, and the weight-bearing force exerted by the hind limb was measured and averaged over a 3 s period. The weight distribution ratio was calculated as described previously [20].

#### 2.2.4. Histopathological Analysis

Following rat sacrifice at 4 weeks, tissue specimens from the knee joint were removed, fixed in 10% formalin, embedded in paraffin, and serially sectioned. Hematoxylin and eosin (H&E) or Safranin O-fast green staining was performed to visualize joint cells and matrices. Histological changes were examined by light microscopy (Olympus CX31/BX51, Olympus Optical Co., Tokyo, Japan) and photographed (Olympus DP70).

#### 2.2.5. Measurement of Serum Cytokine and Inflammatory Mediator Levels

Cytokine levels in serum were measured by centrifuging blood samples (1500× *g*, 10 min, 4 °C), and serum was collected and stored at −70 °C until needed. Levels of tumor necrosis factor (TNF)-α, interleukin (IL)-6, leukotriene B4 (LTB4) and 5-lipoxygenase (5-LOX) in serum were measured using ELISA kits from R&D Systems (Minneapolis, MN, USA) according to the manufacturer’s instructions.

#### 2.2.6. Real-Time Quantitative RT-PCR Analysis

Total RNA was extracted from knee joint tissue using TRIzol reagent (Sigma-Aldrich, Steinheim am Albuch, Germany), reverse-transcribed into cDNA and PCR-amplified using a TM One-Step RT PCR kit with SYBR green reagent (Applied Biosystems, Grand Island, NY, USA). Real-time quantitative PCR was performed using a 7500 Real-Time PCR system (Applied Biosystems, Grand Island, NY, USA). Aliquots of sample cDNAs and an equal amount of GAPDH cDNA were amplified using the TaqMan Universal PCR master mixture containing DNA polymerase according to the manufacturer’s instructions (Applied Biosystems, Foster, CA, USA). PCR amplification cycling conditions were 2 min at 50 °C, 10 min at 94 °C, 15 s at 95 °C, and 1 min at 60 °C for 40 cycles. The relative expression of the target gene was determined using the comparative *C*_t_ (threshold cycle number at the cross-point between amplification plot and threshold) method according to the manufacturer’s instructions. The sequences of primers and probes used are listed in Table 1.

#### 2.2.7. Statistical Analysis

All results are presented as the mean ± standard error of the mean (SEM). Statistical analysis was performed using one-way analysis of variance with Dunnett’s multiple comparisons test for multiple comparisons, and *p* < 0.05 was considered statistically significant. Statistical analysis was performed using GraphPad Prism Software version 6.0 for Windows (GraphPad Software, La Jolla, CA, USA).

### 2.3. Network Pharmacology Analysis

#### 2.3.1. Screening of Active PJ Components

To investigate the holistic characteristics, active components of PJ were identified by searching the literature and public databases PubMed [21], and the Korean Traditional Knowledge Portal [22]. Through this, the PJ components were collected and finally selected as bioactive compounds, isolated or identified compounds from PJ. Chemical structures, synonyms, molecular weight, 2D structure, chemical number and physicochemical properties were confirmed using ChEMBL [23] and PubChem [24].

#### 2.3.2. Pharmacokinetic Absorption, Distribution, Metabolism and Excretion (ADME) Evaluation

PJ compounds (*n* = 103) were selected using in silico integrative oral bioavailability (OB), and drug-likeness (DL) was screened using absorption, distribution, metabolism, and excretion (ADME) models administered by the Traditional Chinese Medicine Systems Pharmacology (TCMSP) Database [25]. Compounds without ADME information were removed. The ADME system used in this study includes predicted OB and DL, and compounds were retained only if OB ≥ 30 and/or DL ≥ 0.18 to satisfy criteria suggested by the TCMSP database [26]. The above criteria were used to select the final set of compounds (*n* = 12; Table S1) as candidates for subsequent analysis. These compounds are the main components/active functional ingredients of PJ [10,11,13,14,27].

#### 2.3.3. Identification of Associated Compounds and Target Genes

To gather information on interactions between PJ compounds and associated genes, the Search Tool for Interactions of Chemicals and Proteins (STITCH) database was used [16,28]. Using this database, chemical-protein interactions with a combined score of ≥400 (as medium confidence) were retained [29,30,31]. Next, the association between these compounds and genes with OA was examined by searching the Therapeutic Targets Database (TTD) [32]. A list of target genes (*n* = 42; Table S2) was then finalized.

#### 2.3.4. Network Construction and Analysis

To investigate interactions between PJ compounds and OA target genes, networks were constructed using network visualization software Cytoscape ver. 3.5.1 [33,34]. This software was used to visualize biological pathways and molecular interaction networks, and for data integration, analysis, and visualization/analysis of complex networks. In networks, nodes represent compounds or target genes, and edges indicate compound-target gene interactions. After network analysis, functional annotation of genes was carried out using the Database for Annotation, Visualization, and Integrated Discovery (DAVID), ver. 6.8 [35] and the Kyoto Encyclopedia of Genes and Genomes (KEGG) [36].

## 3. Results

### 3.1. PJE Administration Restored the Hind Paw Weight-Bearing Distribution in MIA-Induced OA Rats

To assess the safety of PJE administration, the weight of rats was monitored over 4 weeks, and the initial and final weights did not differ among experimental animals (Figure 1B). Weight bearing distribution, as a measure of OA progression and the efficacy of anti-inflammatory compounds, was measured as the difference between sensitized (MIA-injected) and contralateral hind limbs [37,38]. To evaluate the effects of PJE on the progression of OA, we assessed the hind paw weight-bearing capability for 21 days after MIA induction. As shown in Figure 1C, the average weight distribution of the saline group was 50% ± 3% between MIA-sensitized and hind legs. In OA-induced rats, a shift in weight distribution occurred towards contralateral limbs after MIA injection, and joint discomfort remained constant until day 21. On day 7 following MIA injection, the MIA group exhibited a significantly lower weight-bearing distribution, which was maintained for at least 21 days. However, in the PJE-administered group, there was a slight but significant decrease in this ratio relative to controls at day 7, but this gradually increased and recovered after 21 days. These results demonstrate restoration of balance and relief of joint discomfort in the PJE-treated group.

### 3.2. PJE Treatment Recovered the Histopathological Features of Joint Tissue in MIA-Induced OA Rats

**Figure 2:**
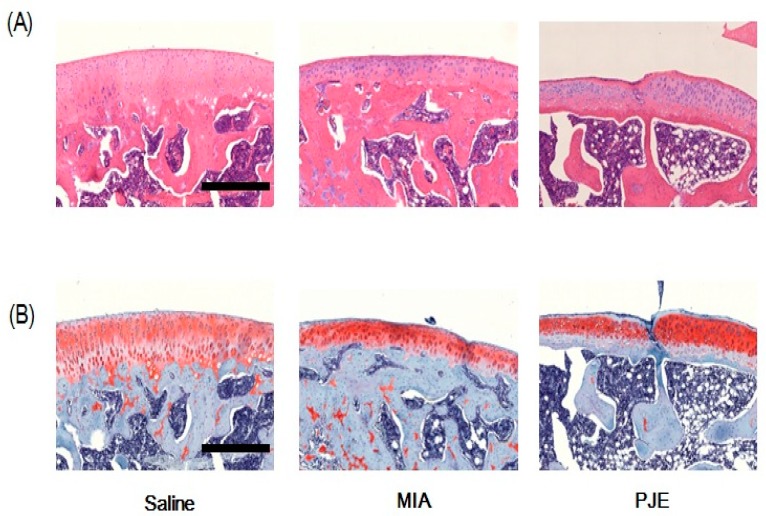
Histopathological features of knee joint tissue of MIA-induced OA model rats. Representative photographs of knee joint tissues stained with (**A**) hematoxylin and eosin, and (**B**) safranin O-fast green (×100 magnification). Scale bars = 500 μm.

Cartilage degradation in animals suffering from OA was observed histopathologically. Thus, we investigated whether PJE could exert a therapeutic effect in vivo using the MIA-induced OA rat model. Bone sections were stained with H&E (Figure 2A) and safranin O/Fast Green (Figure 2B), and MIA-induced OA rats exhibited a greater number of articular chondrocytes, as well as subchondral bone remodeling. Similarly, the number of chondrocytes was increased in the PJE-treated group relative to the control group treated with vehicle. However, the height of cartilage was decreased in the subchondral bone of MIA-induced OA rats, but was increased in the subchondral bone tissue of PJE-treated rats. These histological features indicated that cartilage damage caused by MIA injection was reversed in the PJE-treated group.

### 3.3. PJE Administration Decreased Inflammatory Mediator Levels in Serum

**Figure 3:**
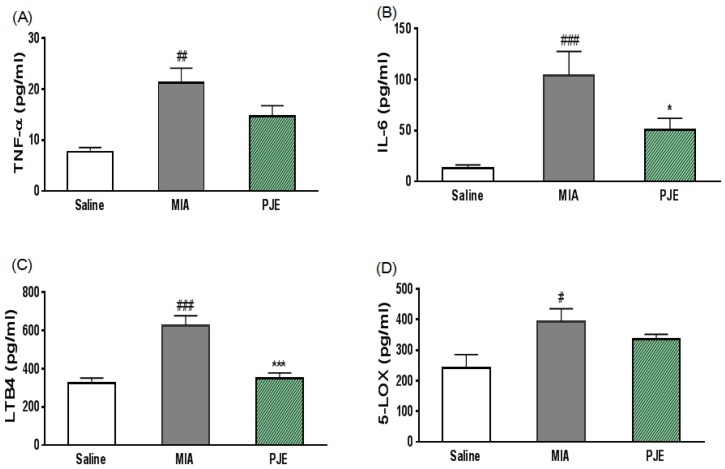
Effects of PJE on serum levels of cytokines and inflammatory mediators in MIA-induced OA model rats. Levels of (**A**) TNF-α: Tumor necrosis factor alpha, (**B**) IL-6: Interleukin 6, (**C**) LTB4: Leukotriene B4, and (**D**) 5-LOX: 5-lipoxygenase in serum measured by ELISA. # *p* < 0.05, ## *p* < 0.01, and ### *p* < 0.001 vs. saline controls; * *p* < 0.05 and *** p < 0.001 vs. the MIA group.

Various inflammatory mediators have been identified in OA and might be involved in pathogenesis [39]. Therefore, we investigated the effects of PJE on serum levels of TNF-α, IL-6, LTB4, and 5-LOX in MIA-induced OA model rats. As shown in Figure 3A–D, compared with the saline control group, the MIA group displayed significantly higher levels of TNF-α, IL-6, LTB4 and 5-LOX. By contrast, levels of IL-6 and LTB4 were lower in the PJE-treated group, and although the difference was not significant, TNF-α and 5-LOX were slightly lower in the PJE group.

### 3.4. PJE Treatment Inhibited mRNA Expression Levels of Inflammatory Mediators in MIA-Induced OA Rats

**Figure 4:**
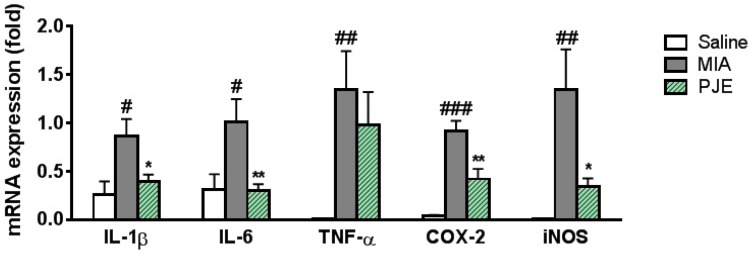
Effects of PJE on the expression of cytokines and inflammatory mediators in the knee joint of MIA-induced OA model rats. Expression of IL-1β, IL-6, TNF-α, COX-2 and iNOS mRNA levels determined by real-time PCR. # *p* < 0.05, ## *p* < 0.01 and ### *p* < 0.001 vs. saline controls; * *p* < 0.05 and ** *p* < 0.01 vs. the MIA group. COX-2, iNOS. IL-1β: Interleukin 1 beta; IL-6: Interleukin 6; TNF-α: Tumor necrosis factor alpha; COX-2: Cyclooxygenase-2; iNOS: Inducible nitric oxide synthases.

OA is a chronic disease affecting joints that results in increased production of various inflammatory mediators, and interactions within the cytokine network are known to be important [39,40]. We therefore examined the effects of PJE on mRNA expression levels of inflammatory mediators (IL-1β, IL-6, TNF-α, COX-2, and iNOS) in the knee joint of MIA-induced rats. As shown in Figure 4, the results showed that levels of all five were significantly increased in the MIA group, but mRNA expression levels of IL-1β, IL-6, COX-2, and iNOS were lower in the PJE-treated group. 

### 3.5. In Silico Network Analysis and Prediction of Target Genes and Pathways Related to OA

**Figure 5:**
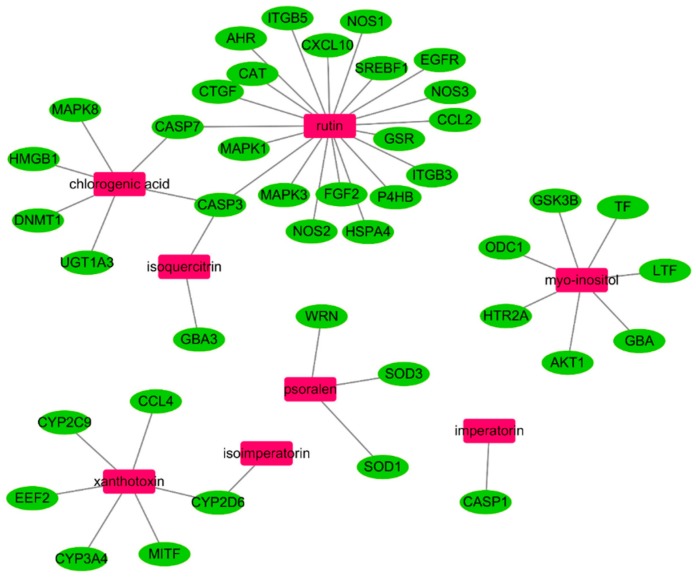
Compound-target gene network linking the protective effects of PJE against OA to potential target genes and signaling pathways. Compounds derived from PJE are indicated by pink rectangles, target genes are represented by green ovals, and gray lines represent compound-target gene interactions. PJE: *Peucedanum japonicum* extract; OA: Osteoarthritis.

To further clarify the interactions between active compounds of PJ and OA target genes, relationships were investigated using network analysis. The resulting network includes eight PJE components and the 42 potential target genes are shown in Figure 5. Most compounds are linked with more than one target, and the main nodes rutin (20), myo-inositol (7), chlorogenic acid (6) and xanthotoxin (6) are linked to six or more genes. In addition, kernel genes, caspase-3 (CASP3; rutin, chlorogenic acid, and isoquercitrin), caspase-7 (CASP7; rutin and chlorogenic acid), and cytochrome P450 2D6 (CYP2D6; xanthotoxin and isoimperatorin), were regulated by two or more compounds in this network. These major compounds and genes are more likely to play important roles in OA disease progression, while others interacting with only one compound are less likely to have a central role.

To better understand the signaling pathways and functions of these target genes, we carried out functional enrichment analysis using DAVID software and the KEGG database; potential target genes were functionally associated with various signal transduction pathways such as Tumor necrosis factor (TNF), Hypoxia-inducible factor 1 (HIF-1), phosphatidylinositol 3' -kinase-Akt (PI3K-Akt), mitogen-activated protein kinase (MAPK), and Vascular endothelial growth factor (VEGF) (Table 2). Interestingly, many of the potential target genes appear to be connected to the TNF signaling pathway, suggesting that this pathway mediates the effects of PJE components against OA.

## 4. Discussion

OA progression and development are complex processes involving multiple factors that alter the homeostasis of chondrocytes. Likewise, multiple compounds contained within herbal medicines can act on multiple target proteins to exert their biological and pharmacological effects in human diseases. To investigate the effects of an herbal medicine for the treatment of OA disease, we attempted to verify the efficacy of PJE using the MIA-induced rat model, and further explored the potential molecular mechanisms of PJE components through a systematic approach using network pharmacology.

Using the OA rat model, our histopathological results showed that PJE exerted potential protective effects against OA. Specifically, we observed changes in weight bearing distribution and levels of inflammatory factors in serum and joint tissue. Inflammatory factors such as cytokines and lipid inflammatory mediators have been implicated in OA pathogenesis. In particular, serum levels of TNF-α and IL-6 have been associated with OA severity, narrowing of joint space and knee cartilage loss. LTB4 increases the production and release of cytokines such as IL-1β and TNF-α by synovial tissues [39,41]. The enzyme COX-2 is upregulated in inflamed joint tissues and is responsible for elevated production of lipid mediators in OA joints. Previous research suggests that overexpression of COX-2 is likely induced by pro-inflammatory mediators including IL-1β, TNF-α and IL-6, and these mediators, as well as 5-LOX, can be employed for assessing the efficacy of OA treatments [42,43,44]. Thus, PJE appears to play an important role in preventing or slowing the progression of OA by regulating inflammatory reactions.

From our network pharmacology analysis, we concluded that the main active compounds in PJE, including rutin, myo-inositol, chlorogenic acid, and xanthotoxin [10,12,14], might play key roles in the treatment of OA by regulating key target genes such as CASP3, CASP7, and CYP2D6. These main nodes, identified by KEGG pathway analysis, are associated with various signaling pathways, and the TNF signaling pathway was found to be strongly linked to OA. Previous studies of the effects of active compounds in OA showed that rutin was found to protect rat articular chondrocytes against oxidative stress induced by hydrogen peroxide via activation of silent information regulator 1 (SIRT1) [45]. Chlorogenic acid exhibits anti-arthritic effects in rabbit chondrocytes and OA models by altering protein expression and reducing cartilage degradation [46]; xanthotoxin suppresses the expression of chondrocyte hypertrophic genes by inactivating the p38-MAPK/HDAC4 signaling pathway [47]. These compounds exerting protective effects in OA are therefore possible treatment candidates.

We identified CASP3 and CASP7 as potential target genes, and these apoptosis markers play a central role in chondrocyte apoptosis, a complex process involving the activation of multiple intercellular signaling pathways including the caspase cascade. The regulatory mechanisms of this process are likely to be valid targets for modulating cartilage degeneration in future OA treatments [48,49]. The CYP2D6 enzyme involved in drug metabolism in the liver reportedly interacts with Tramadol, an analgesic used to treat moderate to moderately severe pain such as that experienced by OA patients [50]; however, further studies on the relevance to OA are needed. The functions of potential target genes identified from KEGG analysis were associated with multiple signal transduction pathways such as TNF, HIF-1, PI3K-Akt, MAPK, and VEGF. These pathways contribute to the complex regulation of OA disease progression, and the therapeutic effects of OA-active compounds. For instance, previous research reported that targeting TNF alleviates the symptoms of inflammatory OA [51]. The HIF-1 signaling pathway has also been linked to OA via the actions of a survival factor that affects autophagy and apoptosis, and hence cartilage homeostasis [52]. In addition, the PI3K/Akt signaling pathway is important for regulating chondrocyte apoptosis [53]. MAPK and VEGF signaling pathways are also involved in OA pathogenesis and could be considered potential OA therapeutic targets [54,55]. Therefore, our results suggest that these pathways might be coordinated during OA disease progression, and the effects of PJE could be mediated through multiple signaling pathways.

## 5. Conclusions

In conclusion, our results revealed that the protective effects of PJE restored the hind paw weight distribution in OA model rats by alleviating the histopathological features in the cartilage and suppressing levels of inflammatory mediators. Our subsequent network analysis identified CASP3, CASP7, and CYP2D6 as potential target genes playing roles in various signaling pathways; the TNF signaling pathway in particular appears to be linked to the therapeutic activity of PJE components in OA rats. Thus, our combined OA animal model and pharmacological network analyses confirmed the beneficial effects of PJE against OA and identified key intracellular signaling pathways connecting active compounds and target genes linked to OA. Further research is needed to identify the individual components responsible for the therapeutic activities, to establish their mechanisms of action, and to confirm the target genes and signaling pathways involved.

## Figures and Tables

**Table 1:** Sequences of real-time PCR primers.

Gene		Primer Sequence	Accession No.
IL-1β	Forward Reverse	5′-CCCTGCAGCTGGAGAGTGTGG-3′ 5′-TGTGCTCTGCTTGAGAGGTGCT-3′	NM_031512.2
IL-6	Forward Reverse	5′-TTCCTACCCCAACTTCCAATG-3′ 5′-ATGAGTTGGATGGTCTTGGTC-3′	NM_012589.1
TNF-α	Forward Reverse	5′-GACCCTCACACTCAGATCATCTTCT-3′ 5′-TGCTACGACGTGGGCTACG-3′	NM_012675.3
COX-2	Forward Reverse	5′-TGGTGCCGGGTCTGATGATG-3′ 5′-GCAATGCGGTTCTGATACTG-3′	S67722.1
iNOS	Forward Reverse	5′-CTTTACGCCACTAACAGTGGCA-3′ 5′-AGTCATGCTTCCCATCGCTC-3′	NM_012611.3
GAPDH	Probe	Applied Biosystems Rat GAPD (GAPDH) Endogenous Control (VIC/MGB Probe, 4352338E)	

IL-1β: Interleukin 1 beta; IL-6: Interleukin 6; TNF-α: Tumor necrosis factor alpha; COX-2: Cyclooxygenase-2; iNOS: Inducible nitric oxide synthases; GAPDH: Glyceraldehyde 3-phosphate dehydrogenase.

**Table 2:** Kyoto Encyclopedia of Genes and Genomes (KEGG) pathways and target genes of compounds in *Peucedanum japonicum* extract (PJE) potentially responsible for the therapeutic activities against osteoarthritis.

Pathway Classification	Pathway ID	Term	Target Gene
Signal transduction	hsa04668	TNF signaling pathway	AKT1, CCL2, CXCL10, CASP3, CASP7, MAPK1, MAPK3, MAPK8
Signal transduction	hsa04066	HIF-1 signaling pathway	AKT1, EGFR, MAPK1, MAPK3, NOS2, NOS3, TF
Signal transduction	has04310	ErbB signaling pathway	AKT1, EGFR, GSK3B, MAPK1, MAPK3, MAPK8
Signal transduction	has04151	PI3K-Akt signaling pathway	AKT1, EGFR, FGF2, GSK3B, ITGB3, ITGB5, MAPK1, MAPK3, NOS3
Signal transduction	hsa04068	FoxO signaling pathway	AKT1, CAT, EGFR, MAPK1, MAPK3, MAPK8
Signal transduction	hsa04010	MAPK signaling pathway	AKT1, CASP3, EGFR, FGF2, MAPK1, MAPK3, MAPK8
Signal transduction	hsa4370	VEGF signaling pathway	AKT1, MAPK1, MAPK3, NOS3
Signal transduction	hsa04015	Rap1 signaling pathway	AKT1, EGFR, FGF2, ITGB3, MAPK1, MAPK3
Signal transduction	hsa04014	Ras signaling pathway	AKT1, EGFR, FGF2, MAPK1, MAPK3, MAPK8
Signal transduction	hsa04020	Calcium signaling pathway	HTR2A, EGFR, NOS1, NOS2, NOS3
Signal transduction	hsa04150	mTOR signaling pathway	AKT1, MAPK1, MAPK3

## Supplementary Materials

The following are available online at http://www.mdpi.com/2072-6643/10/6/754/s1, Table S1: List of 12 active compounds in *Peucedanum japonicum* extract (PJE) passing the absorption, distribution, metabolism and excretion (ADME) screening criteria, Table S2: List of 42 potential osteoarthritis-related target genes.

## Abbreviations

OA	Osteoarthritis
PJ	*Peucedanum japonicum*
PJE	*Peucedanum japonicum* extract
MIA	Monosodium iodoacetate
CASP3	Caspase-3
CASP7	Caspase-7
CYP2D6	Cytochrome P450 2D6
TNF-α	Tumor necrosis factor
IL-6	Interleukin-6
LTB4	Leukotriene B4
5-LOX	5-lipoxygenase
OB	Oral bioavailability
DL	Drug-likeness
ADME	Absorption, distribution, metabolism, and excretion
TCMSP	Traditional Chinese Medicine Systems Pharmacology
STITCH	Search Tool for Interactions of Chemicals and Proteins
TTD	Therapeutic Targets Database
DAVID	Database for Annotation, Visualization, and Integrated Discovery
KEGG	Kyoto Encyclopedia of Genes and Genomes
SIRT1	Silent information regulator 1
