# Supplementary material for: Protective Effects of Peucedanum japonicum Extract against Osteoarthritis in an Animal Model Using a Combined Systems Approach for Compound-Target Prediction
Source: Nutrients. 2018 Jun 11;10(6):754. doi: 10.3390/nu10060754 (PMC6024510; doi:10.3390/nu10060754)
Supplement: Supplementary file 1 [file nutrients-10-00754-s001.pdf]

Supplemental Material: Protective effects of Peucedanum japonicum extract against osteoarthritis in an animal model using a combined systems approach for compound-target prediction

Table S1. List of 12 active compounds in *Peucedanum japonicum* extract (PJE) passing the absorption, distribution, metabolism and excretion (ADME) screening criteria (Supplementary Table S1).

Compound	Structure
(+)-Marmesin	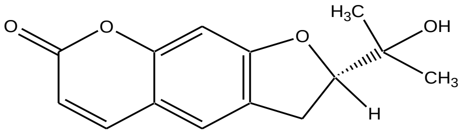
(S)-(+)-2-Methylbutyric acid	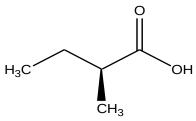
Bergapten	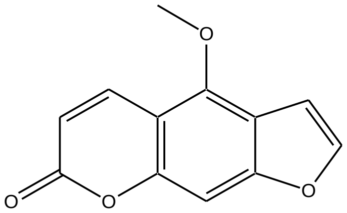
Chlorogenic acid	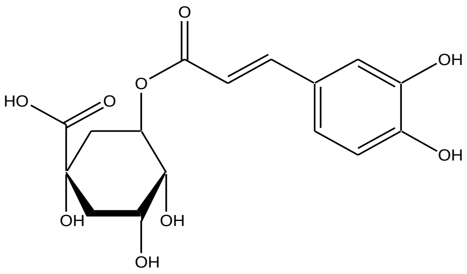
Imperatorin	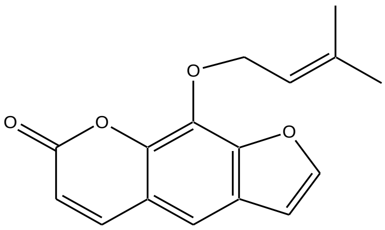
Isoimperatorin	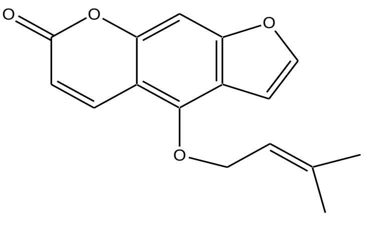

Isoquercitrin	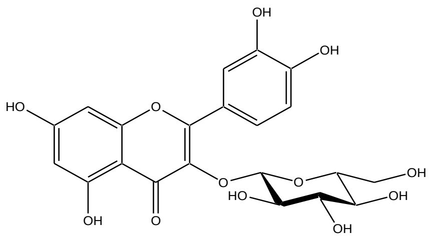
Myo-inositol	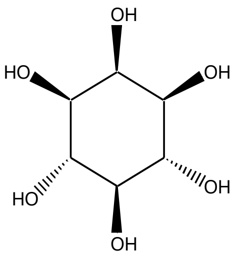
Psoralen	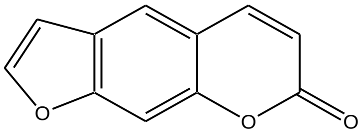
Rutin	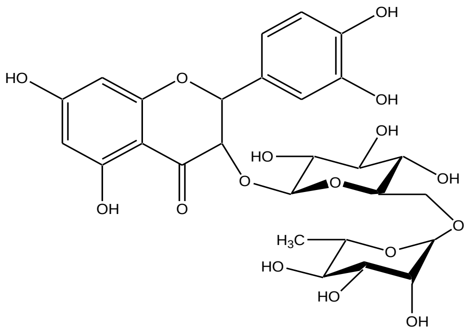
Xanthotoxin	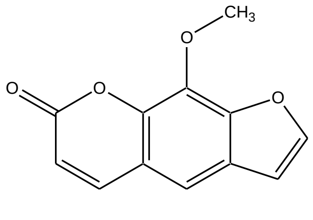
Xanthotoxol	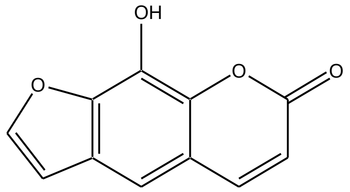

Table S2. List of 42 potential osteoarthritis-related target genes (Supplementary Table S2).

Target Gene	Target Protein
CASP3	caspase-3
MAPK8	mitogen-activated protein kinase 8
DNMT1	DNA methyltransferase 1
HMGB1	high mobility group box 1
UGT1A3	UDP glucuronosyltransferase family 1 member A3

CASP7	caspase-7
CASP1	caspase-1
CYP2D6	cytochrome P450 family 2 subfamily D member 6
GBA	glucosylceramidase beta
AKT1	AKT serine/threonine kinase 1
HTR2A	5-hydroxytryptamine receptor 2A
LTF	lactotransferrin
TF	transferrin
GSK3B	glycogen synthase kinase 3 beta
ODC1	ornithine decarboxylase 1
SOD1	superoxide dismutase 1
WRN	Werner syndrome RecQ like helicase
SOD3	superoxide dismutase 3
P4HB	prolyl 4-hydroxylase subunit beta
EGFR	epidermal growth factor receptor
CTGF	connective tissue growth factor
HSPA4	heat shock protein family A (Hsp70) member 4
SREBF1	sterol regulatory element-binding transcription factor 1
GSR	glutathione-disulphide reductase
FGF2	fibroblast growth factor 2
NOS2	nitric oxide synthase 2
CXCL10	C-X-C motif chemokine ligand 10
AHR	aryl hydrocarbon receptor
CCL2	C-C motif chemokine ligand 2
MAPK1	mitogen-activated protein kinase 1
MAPK3	mitogen-activated protein kinase 3
NOS1	nitric oxide synthase 1
NOS3	nitric oxide synthase 3
ITGB3	integrin subunit beta 3
CAT	catalase
ITGB5	integrin subunit beta 5
CYP3A4	cytochrome P450 family 3 subfamily A member 4
MITF	melanogenesis associated transcription factor
CYP2C9	cytochrome P450 family 2 subfamily C member 9
CCL4	CCL4 C-C motif chemokine ligand 4
EEF2	eukaryotic translation elongation factor 2
GBA3	glucosylceramidase beta 3
